# Two Years after Molecular Diagnosis of Familial Hypercholesterolemia: Majority on Cholesterol-Lowering Treatment but a Minority Reaches Treatment Goal

**DOI:** 10.1371/journal.pone.0009220

**Published:** 2010-02-15

**Authors:** Roeland Huijgen, Iris Kindt, Sjoerd B. J. Verhoeven, Eric J. G. Sijbrands, Maud N. Vissers, John J. P. Kastelein, Barbara A. Hutten

**Affiliations:** 1 Department of Vascular Medicine, Academic Medical Center (AMC), Amsterdam, The Netherlands; 2 Foundation for the Identification of Persons with Inherited Hypercholesterolemia, Amsterdam, The Netherlands; 3 Department of Cardiovascular Genetics and Department of Pharmacology, Vascular and Metabolic Diseases, Erasmus Medical Center, Rotterdam, The Netherlands; 4 Department of Clinical Epidemiology, Biostatistics and Bioinformatics, Academic Medical Center (AMC), Amsterdam, The Netherlands; Leiden University Medical Center, The Netherlands

## Abstract

**Background:**

The risk of premature cardiovascular disease in patients with familial hypercholesterolemia (FH) can be profoundly reduced by cholesterol-lowering therapy, and current guidelines for FH advocate ambitious low-density lipoprotein cholesterol (LDL-C) goals. In the present study, we determined whether these goals are reflected in current clinical practice once FH has been diagnosed.

**Methodology/Principal Findings:**

In 2008, we sent questionnaires to all subjects (aged 18–65 years) who were molecularly diagnosed with FH in the year 2006 through the screening program in the Netherlands. Of these 1062 subjects, 781 completed the questionnaire (46% males; mean age: 42±12 years; mean LDL-C at molecular diagnosis (baseline): 4.1±1.3 mmol/L). The number of persons that used cholesterol-lowering therapy increased from 397 (51%) at baseline to 636 (81%) after diagnosis. Mean treated LDL-C levels decreased significantly to 3.2±1.1 mmol/L two years after diagnosis. Only 22% achieved the LDL-C target level of ≤2.5 mmol/L.

**Conclusions/Significance:**

The proportion of patients using cholesterol-lowering medication was significantly increased after FH diagnosis through genetic cascade screening. The attained LDL-C levels were lower than those reported in previous surveys on medication use in FH, which could reflect the effect of more stringent lipid target levels. However, only a minority of the medication users reached the LDL-C target.

## Introduction

Familial hypercholesterolemia (FH) is an inherited disorder of lipid metabolism that predisposes to severe premature cardiovascular disease (CVD).[1], [2] Cholesterol-lowering therapy can prevent or delay the onset of CVD and premature death in these individuals.[3], [4]

Although FH is relatively common (1∶400 in The Netherlands), many patients are not diagnosed at all or they are identified only after symptomatic onset of CVD.[5] Therefore, a molecular screening program was set up to actively identify all FH patients in the Netherlands.[6] During the last five years, approximately 9000 FH mutation carriers were identified. However, the success of this national screening program in preventing CVD depends also on the acceptance of physicians and patients to start preventive measures, including foremost cholesterol-lowering medication.

In 2001, we demonstrated that 38% of the participants used cholesterol-lowering therapy before the screening compared to 86% two years after the molecular diagnosis.[7] In the years after this survey, new evidence was presented on the benefit of statin treatment. In fact, two large meta-analyses demonstrated a linear relationship between LDL-C levels and the occurrence of coronary heart disease in both primary and secondary prevention of CVD.[8], [9] Additionally, more aggressive treatment was shown to further decrease CVD risk.[10], [11] New treatment options as well as the growing awareness among physicians of the beneficial effect of aggressive LDL-C lowering have resulted in the development of ambitious treatment targets for persons with FH.[12], [13] Dutch guidelines now recommend a LDL-C treatment target of ≤2.5 mmol/L (97 mg/dL) for subjects with FH.[13] These new insights and guidelines may have changed the treatment of FH patients compared to the setting of the previous survey in 2001.[7]

In the present study, we therefore assessed whether the molecular screening program has improved the preventive care for FH. In addition, we analyzed the determinants that predict the decisions about cholesterol-lowering medication once a molecular diagnosis of FH is made.

## Methods

### Ethics Statement

We recruited subjects from the database of the nation-wide cascade screening program for FH in the Netherlands.[6] This program was instituted by the Dutch government and approved and financed by the Ministry of Health. All subjects who consented to participate in the screening for the FH mutation were also asked for written consent for additional scientific studies. For our survey we only approached those who gave written informed consent for additional studies. We received approval to perform such a questionnaire follow-up study by the Medical Ethical Committee of the Academic Medical Center of the University of Amsterdam.

### Patient Selection and Recruitment

Subjects between 18 and 65 years of age were potentially eligible if they were visited in 2006 by a genetic fieldworker, a functional FH mutation was identified, and the lipid profile was assessed. We excluded patients if their address information was incorrect or unavailable or if they had declared that they did not want to be approached for scientific research. All probands with whom family screening was initiated were excluded as well.

We sent a questionnaire by surface mail to all selected FH patients in May 2008. Two months after the questionnaire had been distributed, we sent a reminder to the subjects who had not returned the questionnaire. If we had not received the questionnaire after six months, we contacted a random sample of the non-responder population by telephone in order to elucidate the medication use at follow-up, and inquired after their reasons for non-response. We selected these non-responders per month in which they were diagnosed, and we choose the months January, April, July, and October in 2006. We subsequently phoned until 25 consecutively diagnosed individuals in each period had consented to participate, which resulted in an additional number of 100 participants.

Data on demographics, CVD risk factors, lipid profile and medication use at baseline were extracted from the database of the screening organization.

### Outcome Measures

The primary endpoints were attained LDL-C levels and the difference of the proportions treated persons at molecular diagnosis (‘at baseline’) and on average two years after the molecular diagnosis (‘at follow-up’). The secondary endpoints were the differences in CVD risk factors and lipid profiles between the individuals who were treated or had been treated with cholesterol-lowering drugs and those who were never treated.

When untreated LDL-C levels or recent on-treatment levels were not available, we imputed these LDL-C levels by means of the estimated LDL-C lowering potency of a specific lipid-lowering drug and dose. These potency scores were summarized in a supplementary table (**table S1**). Potency scores for statins were derived from Walma et al.[13] We estimated the potency of ezetimibe as 1.20 based on the approximately 17% additional LDL-C lowering when added to statins.[14] The potency of a resin was estimated as 1.11 and that of fibrates and nicotinic acid derivatives 1.05.[3] We calculated the age and sex specific percentiles for the LDL-C levels for each subject based on reference values of a Caucasian population.[15]

### Statistical Analyses

Differences in continuous and binary variables between subgroups were compared by linear or logistic regression analyses with generalized estimating equations (GEE) in the SAS procedure GENMOD accounting for correlations within families. The association between initiation of treatment since molecular diagnosis and demographics, clinical characteristics, and lipids, was analyzed with logistic regression (GEE) for all individuals who were treatment naïve at baseline. We assessed the relation between treatment potency of the drug regimen used after the molecular diagnosis and demographic, clinical, and lipid characteristics by linear regression analyses (GEE) for all persons that had been treated at baseline and/or during follow-up. We used paired t-tests to compare LDL-C levels and potency of prescribed medication within persons over time, e.g. at baseline and after on average two years of follow-up. Variables with a skewed distribution were log-transformed before analysis. A p-value<0.05 was considered significant. Statistical analyses were performed with the SAS package version 9.1 (SAS Institute Inc, Cary, NC, USA).

## Results

### Study Population

During 2006, a pathogenic LDLR or apolipoprotein B (ApoB) mutation was identified in 341 probands with a clinical diagnosis of FH. In the same period, a total of 4228 relatives aged between 18 and 65 years were screened for the presence of the specific mutation that was identified in the proband. Such a mutation was identified in 1328 of these relatives. Of these mutation carriers, 458 (34%) were first, 257 (19%) were second, 276 (21%) were third and 312 (23%) were fourth or further degree relatives of the proband, and for 25 (2%) we could not retrieve the distance from the proband.

We excluded 266 patients with FH: 134 had not given written informed consent for additional studies at the time of molecular diagnosis and of 132 we lacked up-to-date contact information. Of the remaining 1062 persons, 781 (74%) completed the questionnaire of whom 100 completed it upon a telephone call. The two main reasons for not returning the questionnaire by surface mail were that they had not received the two mailings (n = 33) or that they had been too busy (n = 30).

The 781 participants had a mean age (± standard deviation (SD)) of 42±12 years and a mean LDL-C level at diagnosis of 4.1±1.3 mmol/L and 359 (46%) were male (**table 1**). The overall prevalence of CVD was 10% (n = 77). Most participants had a LDL-receptor mutation (n = 681; 87%). Of the 100 participants with an ApoB mutation, 85 had the R3500Q mutation.[16]

**Table 1:** aracteristics of treated and untreated FH patients.

	Treated n = 636	Untreatedn = 145	p-value
**Demographic and clinical**			
Age *years*	44±12	35±12	<0.001
Male gender *- n (%)*	297 (47)	62 (42)	0.27
History of CVD - *n (%)*	76 (12)	1 (1)	0.002
Diabetes mellitus - *n (%)*	20 (3)	0 (0)	0.02
Hypertension - *n (%)*	75 (12)	2 (1)	<0.001
Body mass index *kg/m^2^*	25±4.4	24±3.8	<0.001
Current smoker (at diagnosis) - *n (%)*	277 (44)	52 (35)	0.05
**Mutation**			
LDLR or ApoB – *n (LDLR)/n (ApoB)*	560/76	121/24	0.10
**Lipids ** ***mmol/L***			
Untreated LDL-C^#^	6.1±2.1	3.9±1.3	<0.001
Percentile untreated LDL-C^#^	92±13	68±29	<0.001
Cholesterol at baseline			
- TC	6.0±1.4^*^	5.7±1.4	0.04
- LDL-C	4.2±1.3^*^	3.9±1.3	0.08
- HDL-C	1.2±0.35^*^	1.3±0.34	0.18
- Triglycerides - *median [IQR]*	1.1 [0.76–1.7]^*^	1.0 [0.64–1.4]	0.006
Reported TC at follow-up	5.2±1.1^a^	5.3±1.3^b^	0.45
Reported LDL-C at follow-up	3.2±1.1^c^	3.4±1.1^d^	0.36
**Treatment**			
Potency of medication at baseline^e^	1.84±0.34^e^	–	–
Potency of medication at follow-up	1.90±0.35	–	–

Data are expressed as mean ± standard deviation unless otherwise indicated. Variables are based on information at time of molecular FH diagnosis in 2006 (baseline) unless otherwise indicated. Follow-up is at completion of the questionnaire in 2008.

ApoB = apolipoprotein B; CVD = cardiovascular disease; HDL-C = high-density lipoprotein cholesterol; IQR = interquartile range; LDL-C = low-density lipoprotein cholesterol; LDLR = LDL-receptor; TC = total cholesterol. ^#^Estimated LDL-C with correction for treatment potency at diagnosis when applicable; ^*^Based on mean LDL-C of treated (n = 397) and untreated (n = 239) levels; ^a^n = 441; ^b^n = 51; ^c^n = 262; ^d^n = 35, ^e^n = 397.

The 281 non-participants, i.e. those who did not respond to the questionnaires and could not be reached by telephone, were significantly younger (36 vs. 42 years of age; p<0.001), had a lower prevalence of CVD (4% vs. 10%; p = 0.003), were more often smokers (56% vs. 42%; p<0.001) and used less cholesterol-lowering medication at baseline (37% vs. 51%; p<0.001) than the 781 participants.

### Use of Cholesterol-Lowering Medication

**Figure 1:**
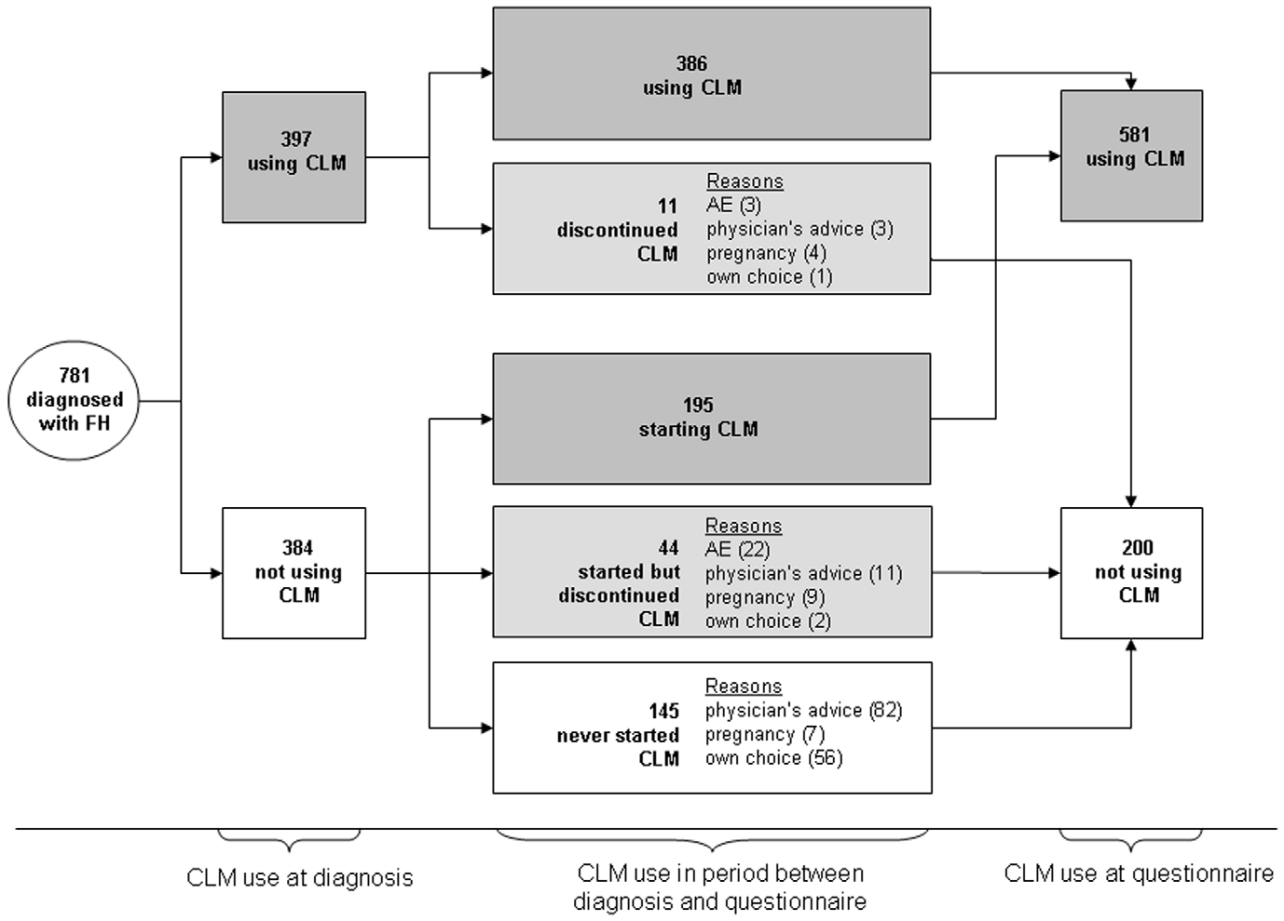
Use of cholesterol-lowering medication between molecular diagnosis of FH and completion of questionnaire. Molecular diagnosis of FH was made in 2006 and the questionnaire was send and completed in 2008. AE = adverse events related to CLM; FH = familial hypercholesterolemia; CLM = cholesterol-lowering medication; pregnancy = no use of medication due to pregnancy, intention to become pregnant or breastfeeding.

At baseline, 397 of the 781 subjects (51%) already used cholesterol-lowering medication and 239 of 384 initially untreated persons (62%) started cholesterol-lowering treatment during follow-up (**figure 1**). Hence, the total number of treated persons increased from 397 (51%) at baseline to 636 (81%) during follow-up (p<0.001). As expected, the group that initiated treatment before the molecular diagnosis maintained treatment much better compared to those starting during follow-up (97% vs. 81%, respectively; p<0.001). During follow-up, a total of 55 patients discontinued medication for various reasons: adverse events (n = 25; 45%); physician's advice (n = 14; 25%); intention to become pregnant, pregnancy or to breastfeed (n = 13; 24%); and own choice (n = 3; 5%).

Nearly one fifth (n = 145) of the 781 participants never started medication. The main reasons were physicians' advice (n = 82; 56%); own choice (n = 56; 39%); intention to become pregnant, pregnancy or to breastfeed (n = 7; 5%).

### LDL-C Levels at Molecular Diagnosis and Two Years after Diagnosis

After two years of follow-up, the mean LDL-C level (± SD [interquartile range (IQR)]) of all participants, was significantly reduced by 22% as compared with the level at baseline (3.2±1.1 [2.6–3.8] vs. 4.1±1.3 [3.2–4.9] mmol/L, respectively; p<0.001). In the group of 239 treatment naïve persons that started cholesterol-lowering medication after diagnosis the mean LDL-C level (± SD) was decreased with 44% from 5.1±1.3 at baseline to 2.8±0.80 mmol/L (p<0.001) two years later. The mean additional reduction in patients who were already treated at baseline was 8%: from 3.5±0.9 to 3.2±0.9 mmol/L (p<0.001).

The mean baseline LDL-C levels ± SD [IQR] differed significantly between 239 treatment naïve subjects at diagnosis who started medication during follow-up and 145 subjects who did not initiate treatment (5.1±1.3 [4.2–5.7] vs. 3.9±1.3 [3.0–4.8] mmol/L, respectively; p<0.001). The mean age and sex specific percentiles of the untreated LDL-C levels were considerably higher in the subjects that initiated medication after the molecular diagnosis than those who did not (88th vs 68th percentile, respectively; p<0.001).

### Characteristics of Treated and Untreated Individuals

The 636 persons, who already used medication at baseline or started during follow-up (i.e. ‘treated’), were older (44±12 vs. 35±12 years; p<0.001) than those 145 persons, who had never used medication (i.e. ‘untreated’) (see **table 1**). As expected, the treated patients had a higher prevalence of cardiovascular risk factors than untreated patients, such as history of CVD, diabetes mellitus, hypertension and history of smoking.

The proportion of ApoB mutation carriers among treated subjects did not differ significantly from the proportion among untreated subjects at follow-up (12% vs. 17%, respectively; p = 0.10). The four most prevalent LDLR mutations that had been reported to result in a severe LDL-C and CVD phenotype, i.e. V408M (exon 9), 1359-1 (intron 9), 313+1/2 (intron 3) and W23X (exon 2), were more prevalent in the treated than in the untreated group (97/636 = 15% vs. 7/145 = 5%; p<0.001).[17] The treated carriers were more often first degree relatives of the proband as compared to the non-treated carriers (231/636 = 36% vs. 40/145 = 28%, p<0.001).

Treated patients (n = 581) had more frequently been referred to a specialist than untreated patients: 312 (54%) visited an internist and/or a cardiologist, whereas 26 of the 145 untreated patients (18%) had visited a specialist. In fact, 65 (45%) of the untreated patients responded that they did not visit a doctor at all with regard to FH treatment.

### Predictors of Initiation of Treatment

Among the 384 untreated subjects at baseline, increased age, body mass index (BMI), LDL-C and triglycerides levels and low levels of high-density lipoprotein cholesterol (HDL-C) were associated with the start of medication after molecular diagnosis in univariate analyses. After stepwise multivariate regression analysis, age, LDL-C, and HDL-C independently predicted the start of medication (**table 2**).

**Table 2:** Association of CVD risk factors with initiation of cholesterol-lowering medication in FH patients that were untreated at diagnosis (n = 384).

	Univariate		Multivariate	
	OR [95% CI]	p-value	OR [95% CI]	p-value
Age *years*	1.03 [1.01–1.05]	0.0060	1.02 [1.0009 – 1.05]	0.041
Male gender	1.30 [0.88–1.92]	0.18	–	–
History of CVD	1.07 [0.09–13.4]	0.96	–	–
Hypertension	3.11 [0.81–12.0]	0.10	–	–
Body Mass Index *kg/m^2^*	1.07 [1.01–1.12]	0.017	–	–
Smoker at diagnosis	1.29 [0.90–1.83]	0.16	–	–
LDL-C *mmol/L*	2.11 [1.65–2.71]	<0.001	2.03 [1.60–2.58]	<0.001
HDL-C *mmol/L*	0.48 [0.27–0.86]	0.014	0.45 [0.22–0.93]	0.031
Triglycerides^#^ *mmol/L*	1.69 [1.19–2.40]	0.0033	–	–

CVD = cardiovascular disease; HDL-C = high-density lipoprotein cholesterol; LDL-C = low-density lipoprotein cholesterol; OR = odds ratio. ^#^log-transformed before analyses.

### Characteristics of Used Cholesterol-Lowering Treatment

The mean potency of the medication of the 636 treated patients was 1.90±0.35. For the 397 subjects that already used cholesterol-lowering medication at baseline, the LDL-C lowering capacity was increased after the identification of their mutation from 1.84±0.34 to 1.99±0.36 (p<0. 001). A total of 265 (34%) participants used dairy products enriched with sterols and stanols, with a similar proportion in those did or did not use cholesterol-lowering medication (34% vs. 33%, respectively; p = 0.92).

### Predictors of Potency of Treatment

For the 636 persons that used cholesterol-lowering medication during follow-up, a number of variables were significantly associated in the univariate analyses with the potency of the drug regimen after diagnosis: increased age and BMI, history of CVD, presence of diabetes or hypertension, smoking status at diagnosis, high pre-treatment LDL-C and triglycerides, and low HDL-C. After stepwise multivariate regression analysis, untreated LDL-C levels, age, history of CVD, and diabetes were independent predictors of the potency of the drug regimen used after the molecular diagnosis (**table 3**).

**Table 3:** Association of CVD risk factors with the potency of cholesterol-lowering drug regimen in all treated FH patients (n = 636).

	Univariate		Multivariate	
	B [95% CI]	p-value	B [95% CI]	p-value
Age *years*	0.0058 [0.10; 0.15]	<0.001	0.0030 [0.0009; 0.0052]	0.0057
Male gender	0.016 [−0.034; 0.066]	0.53	–	–
History of CVD	0.22 [0.14; 0.31]	<0.001	0.098 [0.02; 1.2]	0.019
Diabetes	0.23 [0.06; 0.41]	0.008	0.16 [0.02; 0.30]	0.030
Hypertension	0.16 [0.08;0.25]	<0.001	–	–
Body Mass Index *kg/m^2^*	0.0091 [0.003; 0.015]	0.0031	–	–
Smoker at diagnosis	0.070 [0.02;0.12]	0.0089	–	–
Untreat. LDL-C *mmol/L*	0.071 [0.056; 0.086]	<0.001	0.066 [0.054; 0.078]	<0.001
HDL-C *mmol/L*	−0.089 [−0.17; −0.013]	0.022	–	–
Triglycerides^#^ *mmol/L*	0.053 [0.001; 0.10]	0.046	–	–

Untreat. LDL-C =  LDL-C level at FH diagnosis, when applicable corrected for lipid- lowering therapy use to calculate a pre-treatment value. CVD = cardiovascular disease; HDL-C = high-density lipoprotein cholesterol; LDL-C = low-density lipoprotein cholesterol. ^#^log-transformed before analyses.

### LDL-C Target Attainment

Of the 781 participants, 297 (38%) persons could report a LDL-C level at the end of follow-up: the target level of ≤2.5 mmol/L was achieved in 65 (22%) persons. Based on the mean potency of the current medication (1.90), we expected that 25% of the 636 treated patients would attain the target LDL-C level. In total, 492 of the 636 (77%) subjects that used cholesterol-lowering medication after diagnosis did not achieve LDL-C levels ≤2.5 mmol/L. Of these, 176 (36%) did achieve a reduction of 50% or more in LDL-C levels. Of the 145 subjects that remained untreated, 15 (10%) already had LDL-C levels ≤2.5 mmol/L at screening.

## Discussion

In the current study, we found that mean LDL-C levels of individuals, who were untreated at baseline, decreased by 44% with treatment in the two years after molecular diagnosis. This means that cascade screening by molecular diagnosis not only leads to identification of FH but also supports the decision to treat these new FH patients. The decision whether or not to initiate lipid-lowering treatment was found to be dependent on age, and HDL-C and LDL-C levels. The proportion of individuals on cholesterol-lowering medication increased from 51% to 81% during follow-up. In contrast, one fifth of the identified FH subjects never started cholesterol-lowering medication. In the majority of these cases the general practitioner advised to refrain from medication because their untreated LDL-C levels were not so severely increased, i.e. on average at the 68^th^ percentile, compared to the treated persons, whose levels were at the age and sex specific 92^nd^ percentile before initiation of cholesterol-lowering drugs.

The achieved LDL-C level in all participants two years after molecular diagnosis was significantly lower than the achieved level in a similar survey in 2001: 3.2 mmol/L in 2008 vs. 4.2 mmol/L in 2001.[7] This is likely a consequence of the more stringent LDL-C targets for the FH population. Thus, ambitious goals and treatment possibilities for patients with FH indeed seem to be reflected by current clinical practice.

Surveys with a similar topic have also been performed in Norway and the United Kingdom. The survey in Norway focussed on medication use after molecular FH diagnosis among first degree relatives of probands.[18] The proportion of treated adult subjects increased from 67.5% at the time of genetic testing to 86.0% after six months. The decrease in LDL-C was 13.9% for the entire group with a mean achieved LDL-C level of 4.3±1.4 mmol/L at 6 months follow-up. By contrast, in our survey, we found significant lower achieved LDL-C levels (mean 3.2±1.1 mmol/L). This difference may be explained by a lower baseline LDL-C level in our cohort, a longer follow-up period to up-titrate the treatment and a more potent treatment regimen than in the Norwegian survey.

Audits on management of FH patients in outpatient clinics specifically were recently performed in the United Kingdom (UK) and the Netherlands. In the UK, Hadfield and colleagues showed that the proportion of patients on cholesterol-lowering medication ranged between of 88-94% with on-treatment LDL-C levels between 3.0 and 3.7 mmol/L.[19] Similarly, Pijlman and colleagues found mean achieved LDL-C levels of 3.2±1.1 mmol/L with 96% of the patients being on statin treatment for those visiting outpatient clinics in the Netherlands since 2006. [20] In our study, the percentage of subjects on lipid-lowering medication who visited a specialist amounted 93%, which is rather similar to the percentages found by Pijlman et al. and Hadfield et al. and much higher than the average percentage (81%) in our entire cohort. Taken together, one could argue that patients with a molecular diagnosis are better off when referred to a specialized clinic. On the other hand, the FH population that remained at the general practitioner's office clearly had a different phenotype with less raised LDL-C and fewer risk factors.

In general, management of FH patients is not successful when measured against the new guideline targets. In fact, a sizable subset of persons with FH does not get pharmacological treatment at all and the treatment target of LDL-C levels ≤2.5 mmol/L is achieved only in 22% of the treated patients.[13] This failure to meet LDL-C targets may be partly due to the hesitation of some physicians and patients to use most potent available drug regimens. This notion is supported by the findings of Pijlman and colleagues, who conducted a cross-sectional study in five large outpatient lipid clinics in The Netherlands. The main reason why treating physicians did not prescribe maximum therapy to FH patients despite an LDL-C >2.5 mmol/L, was because they accepted that higher LDL-C level.[20] Another reason for not reaching target levels is that some patients may not have been able to due to extremely high LDL-C levels. In our study we found that, of all treated patients who were not on target (LDL-C≤2.5 mmol/L), 36% had LDL-C levels that were reduced by more than 50%. Nonetheless, LDL-C levels could have been further reduced, since only a minority (5%) of all participants was treated with potent dual therapy with the highest dose of atorvastatin or rosuvastatin in combination with ezetimibe. The estimated percentage of subjects that would have achieved an LDL-C level ≤2.5 mmol/L would be 61% based on that most potent lipid-lowering strategy. Thus, even with the most potent dual treatment regimens, in some cases the Dutch target level can not be reached. More potent options are needed.

One fifth of individuals with a molecular diagnosis of FH did not use cholesterol-lowering medication at all. Almost half of the untreated patients had not consulted a physician, which is unexpected, since genetic field workers encouraged all subjects who were found to have dyslipidemia at screening to visit their physician. Moreover, those with confirmed molecular diagnosis were explicitly urged to seek for medical consultation. The other half did not start treatment even though they had consulted a physician, which is remarkable. However, the FH patients who remained untreated had on average few cardiovascular risk factors and above all, absence of severe dyslipidemia. Whether this group of FH ‘patients’ should receive medication based on mutation carriership or not, is of scientific and clinical importance, and should be further elucidated.[21], [22]

It is intriguing to observe that a relatively large group of subjects had a molecular diagnosis of FH but lacked a severe dyslipidemia phenotype. One of the explanations could be a high prevalence in this group of mild mutations such as ApoB mutations, which in general result in a less severe FH phenotype than LDLR mutations.[23] Indeed, we observe a non-significant trend towards a higher prevalence of ApoB among those without severe dyslipidemia in our study population. Conversely, the LDLR mutations reported to result in a severe FH phenotype, are less common in this group.[17] We further speculate that some sequence variants, which were used in the cascade screening programme in 2006 and assumed to cause FH, may not be pathogenic after all. This could have explained the lack of dyslipidemia in some subjects identified with molecular FH in 2006. An ongoing project aims to correctly label such sequence variants as non-pathogenic.[24] A final possible explanation is that other genetic variants counterbalanced the effect of the FH mutation, such as concurrent ApoB or loss-of-function PCSK9 mutations that usually result in hypobetalipoproteinemia.[25]–[27] We aim to perform additional studies in the near future to find out whether the proposed putative explanations hold true.

One drawback of our approach is the possible bias inflicted by non-participation. Based on the characteristics of the 281 patients who did not participate, i.e. lower proportion of cholesterol-lowering medication use at baseline, younger age and lower prevalence of CVD as compared to the 781 participants, one could reason that the proportion of treated subjects at follow-up will be an overestimation. However, LDL-C levels and the proportion of subjects that used of cholesterol-lowering medication at follow-up did not differ between the 681 participants that returned the questionnaire by surface mail and the 100 subjects that completed the questionnaire by telephone. Assuming that these 100 participants by telephone contact are a random sample of the source population of which the 281 non-participants are a part, we expect the impact of potential selection bias to be modest.

In conclusion, the molecular diagnosis of FH leads to an increased proportion of patients that start or intensify cholesterol-lowering medication, and consequently, to a robust decrease in LDL-C levels. The attained LDL-C levels are lower than those reported in a previous survey which could reflect the effect of more stringent lipid target levels. However, only a minority of the patients was treated with a potent drug regimen to reach set targets.

## Supporting Information

Table S1 Potency scoring for several cholesterol-lowering drugs. *Potency scores for statins were derived from Walma Ned Tijdschr Geneeskd 2006;150:18–23, who based the scores themselves on Law BMJ 2003;326:1423-7. #Correction factor for ezetimibe was based on 17% additional decrease in LDL-C (100%/(100%-17%) = 1.20) when added to statins (Kastelein NEJM 2008; 358: 1431-43). Our study population used relative low doses of bile acid sequestrants and these were estimated to have only a modest effect on LDL-C levels. Even lower potency scores were applied for fibrates and nicotinic acid, which influence primarily triglyceride and HDL-cholesterol levels respectively and have a modest effect on LDL-C levels (Huijgen Expert Rev Cardiovasc Ther 2008;6:567-81).
(0.05 MB DOC)
