# Supplementary material for: Two Years after Molecular Diagnosis of Familial Hypercholesterolemia: Majority on Cholesterol-Lowering Treatment but a Minority Reaches Treatment Goal
Source: PLoS One. 2010 Feb 15;5(2):e9220. doi: 10.1371/journal.pone.0009220 (PMC2821409; doi:10.1371/journal.pone.0009220)
Supplement: Table S1 — Potency scoring for several cholesterol-lowering drugs. *Potency scores for statins were derived from Walma Ned Tijdschr Geneeskd 2006;150:18–23, who based the scores themselves on Law BMJ 2003;326:1423-7. #Correction factor for ezetimibe was based on 17% additional decrease in LDL-C (100%/(100%-17%) = 1.20) when added to statins (Kastelein NEJM 2008; 358: 1431-43). Our study population used relative low doses of bile acid sequestrants and these were estimated to have only a modest effect on LDL-C levels. Even lower potency scores were applied for fibrates and nicotinic acid, which influence primarily triglyceride and HDL-cholesterol levels respectively and have a modest effect on LDL-C levels (Huijgen Expert Rev Cardiovasc Ther 2008;6:567-81). (0.05 MB DOC) [file pone.0009220.s001.doc]

*Table S1:* **Potency scoring for several cholesterol-lowering drugs**

| **Drug** | **Dose in mg/day** | **Potency** |
| --- | --- | --- |
| *Statins** |  |  |
| atorvastatin | 10 | 1.60 |
|  | 20 | 1.80 |
|  | 40 | 2.00 |
|  | 80 | 2.20 |
| fluvastatin | 10 | 1.20 |
|  | 20 | 1.30 |
|  | 40 | 1.40 |
|  | 80 | 1.50 |
| pravastatin | 10 | 1.30 |
|  | 20 | 1.30 |
|  | 40 | 1.40 |
|  | 80 | 1.50 |
| rosuvastatin | 10 | 1.80 |
|  | 20 | 1.90 |
|  | 40 | 2.10 |
|  | 80 | 2.40 |
| simvastatin | 10 | 1.40 |
|  | 20 | 1.50 |
|  | 40 | 1.60 |
|  | 80 | 1.70 |
| *Non-statins* |  |  |
| ezetimibe^#^ | 10 | 1.20 |
| bile acid sequestrants | all doses | 1.11 |
| fibrates | all doses | 1.05 |
| nicotinic acid derivatives | all doses | 1.05 |
|  |  |  |

*Potency scores for statins were derived from Walma *Ned Tijdschr Geneeskd* 2006;150:18-23, who based the scores themselves on Law *BMJ* 2003;326:1423-7. ^#^Correction factor for ezetimibe was based on 17% additional decrease in LDL-C (100%/(100%-17%)=1.20) when added to statins (Kastelein *NEJM* 2008; 358: 1431-43). Our study population used relative low doses of bile acid sequestrants and these were estimated to have only a modest effect on LDL-C levels. Even lower potency scores were applied for fibrates and nicotinic acid, which influence primarily triglyceride and HDL-cholesterol levels respectively and have a modest effect on LDL-C levels (Huijgen *Expert Rev Cardiovasc Ther* 2008;6:567-81).
